# Remarks on Syphilitic Inoculation

**Published:** 1866-12

**Authors:** B. L. Bidenkap

**Affiliations:** Christiania


### SELECTIONS.

*Remarks on Syphilitic Inoculation.* B. L. Bidenkap, M. D., of Christiania.

In a series of letters published in *The Lancet*, Mr. Henry Lee has laid before the profession his views of modern syphilidology. As Mr. Lee has done me the honor of mentioning my name in connexion with some of the facts produced, and as I flatter myself to have contributed in part to the development which this branch of medical knowledge is undergoing just now, I feel myself compelled to correct some of the facts on which Mr. Lee has built up his peculiar views, and to make English readers acquainted with some points which apparently have escaped the attention of the above named author.

1. Mr. Lee seems not to have been thoroughly acquainted with the manner in which others have conducted their experiments in testing the auto-inoculability of the indurated syphilitic chancre. He inoculates a few times, and then leaves off, confidently asserting that the chancre in question is non-inoculable. Others, among them myself, and lately Mr. Walter Coulson, repeat the inoculations for weeks. In this manner at last the characteristic pustule will make its appearance, with its consequence—the excavated ulcer. If Mr. Lee had known the facts laid down in literature, he would have known this and a great deal more concerning syphilitic inoculation, which numerous experiments have taught us, and which he would have done well to try for himself. As the case now stands, Mr. Lee has only succeeded in showing the profession that his own experiments have failed because they have not been conducted in the proper manner.

2. The same remarks will apply to the experiments of the Italian surgeon, Dr. Amilcare Ricordi, whose pamphlet Mr. Lee has quoted in favor of his own opinions. By perusing this pamphlet attentively, Mr. Lee would have found that the Italian surgeon has proceeded in the same manner as himself.

3. Mr. Lee has quoted from Dr. Ricordi an account of my experiments in inoculating the matter of indurated sores in Paris, at the Hôpital du Midi, under the observation of M.

Follin. It is said that when I succeeded, it was because I persisted in inoculating from a sore to which M. Follin objected, because he considered it to be "*mixed.*" Mr. Lee would have done better not to quote, in a scientific lecture, what the said Dr. Ricordi heard from Dr. Pellizzari, who had heard it from Dr. Bargioni, who again had it from M. Follin and his interne. Such evidence is like an old tradition, and is not a fit basis on which to erect a scientific conclusion. It is of the same hearsay kind which Dr. Lane has complained of in Mr. Lee's account of the proceedings at the Lock Hospital.

Now, it is a very mild expression to say that this account of what passed in M. Follin's service at the Hôpital du Midi is not an exact statement of facts. To avoid more misrepresentations in future, I feel myself bound to state shortly what passed.

Five patients were pointed out to me by M. Follin as laboring under true, uncomplicated, unmixed, indurated chancre. M. Follin caused these five chancres to be dressed with savine powder, in order to produce suppuration, which did not exist previously. He asked me then to inoculate the secretion which this remedy had produced. The matter from two of these chancres produced characteristic pustules. From the third the result was doubtful. The last two furnished no inoculable matter—probably because they were very old; and only a very slight effect was produced by the savine powder. After M. Follin had seen the positive result of the inoculations—*but not before*—he pronounced the first two which furnished the inoculable matter to be "mixed," and the experiments consequently of no value. I do not doubt in the least that he believed it, because an irritated chancre often assumes this aspect; I only wonder that he did not tell me before. One more patient was pointed out to me as a fit subject for the experiment, but he left the hospital the day following the inoculation. Another, the seventh, was at least selected as the bearer of a typical indurated chancre, and savine powder applied, but for some days no suppuration could be produced. This is a very common thing, as every experimenter ought to know; and in this instance it was the less surprising, as the patient himself constantly washed away the savine powder, and applied the usual *vin aromatique* to the chancre. As might be supposed, the slight mucopurulent discharge from the surrounding parts of the glans and the prepuce which covered the chancre did not produce any effect when inoculated. It seems that M. Follin lost his patience with what he probably thought a very tedious and uninteresting proceeding, and he assured me very politely that he had had enough of it, just on the same day that a small ulceration had formed on the chancre. Of course I could not insist on carrying on the experiments under these circumstances. I too had had enough of it, and I did not think it worth my while to try to convince a man whose opinions, it was clear, were fixed beforehand; and I had seen enough of the manner in which experiments were conducted in that place, to remark the difference between it and the laborious way I had followed hitherto. These are the experiments to which Mr. Lee has alluded. The worst that can fairly be said of them is that they were incomplete, and therefore inconclusive.

4. Mr. Lee refers to three patients whom he has seen, after they had undergone syphilization in Christiana, and he appears to doubt whether any of them had really suffered from syphilis at all. The third case he speaks of as doubtful, because in its early stage it did not follow the course which syphilitic infection usually does. On this case it is right that I should speak, because the patient was originally under my care, and I probably am the only person, except the patient himself, who knows the particulars of his case. The patient, with whom I was personally acquainted, came to me in January, 1865. He had then a small elevated red spot on the mucous membrane of the urethra, just inside the orifice. No distinct hardness could be felt; the centre of this spot had a yellow color corresponding in size to the head of a pin. This spot and the yellow centre gradually enlarged, and at last a superficial ulcer was formed. About a week afterwards several small ulcers formed on the glans and perpuce, and took the course described by M. Auzias Turenne, and related by Mr. Lee. I did not hesitate to foretell the probable consequence—viz: constitutional infection, which had not yet manifested itself when I left Paris at the end of February, and handed the patient over to M. Auzias Turenne. I saw the patient again in Norway in the middle of April. He had then a strongly marked roseola, elevated copper-colored spots on the forehead, and white characteristic plaques on the mucous membrane of the throat. I think this should be enough to convince any one; and I also think it a strong point in favor of syphilization that Mr. Lee failed to detect any trace of syphilis in this patient after his treatment.

5. Mr. Lee, in speaking of the immunity obtained by syphilization, tries to explain the fact by comparing it with an alleged immunity arising from the effects of irritating substances applied to the skin. Now this last immunity or want of reaction is not, as Mr. Lee seems to think, a very common thing, unless the constitution of the patient has been weakened by the repeated applications of such irritants, or by starving, low diet, or other circumstances producing general debility. Under such circumstances there is often a marked want of reaction even against the syphilitic virus, as Mr. Lee will find fully detailed in the writings of Professor Boeck and myself. But, except in debilitated persons, common irritants do not produce such immunity as the chancrous matter, in proof of which I need only point to the large number of cases treated with anatomical plaster, in Christiania, in whom in no single instance did it occur. And general debility is not produced by syphilization, as Mr. Lee would have known if he had watched the progress of the cure.

6. Mr. Lee's ideas of the natural process through which the system is liberated from the effect of morbid agencies are somewhat too rudely shaped to be taken seriously. Professor Virchow, of Berlin, has very ably pointed out that the specific processes which manifest themselves in the syphilitic organism are not the result of simple deposits of morbid matter. They are either inflammatory, forming a hyperplastic process, or more specific, but still of an active kind, more like neoplasmata (granular tumours) than anything else. Likewise the specific artificial ulcers made in syphilization are not simple draining tubes, but affect the system in a specific way. Nobody would think of substituting an antimonial plaster for a vaccine pustule, and there is no more analogy between the action of this remedy and cow-pox, than between it and syphilization. Whether the immunity produced by the syphilization is complete or not is nothing to the point. The fact is that it produces a state of system in which the production of inoculable matter is failing or incomplete. This is enough, both according to theory and experience. The immunity produced by cow-pox, and even by small-pox, is neither absolute nor everlasting, yet no one denies its existence.

7. When Mr. Lee tries to find an explanation of the successful inoculations in an alleged impurity of the lancet, I can only assure him that I always clean my lancet well, and that I, from the beginning of my experiments, have had my attention fixed to this point.

8. The fearful mortality of which Mr. Lee speaks, in consequence of two deaths which have lately occurred in patients who have undergone syphilization in London, does not exist in reality. Why, when he spoke of these, did he make no mention ot the large number of cases treated in the hospital of Christiania? and why did he make no mention of the real cause of death in those two patients? It would appear that he knew no more of the nineteen or twenty cases treated in London than of the 318 cases of which he could have obtained information from my pamphlet, "Apercu des differents Mèthodes employès â l'Hôpital de Christiania, &c.," 1863. Of these 318 cases, with the exception of some children laboring under hereditary syphilis, only two persons have died. The one died of dysentery, the other of puerperal fever.

9. The chief objections of Mr. Lee against syphilization seem to be—1st, that the calomel vapor bath is better; and 2nd, that syphilization is difficult to apply in private practice. With regard to the calomel vapor bath, it is difficult to understand why it should be so tar superior to all other treatments which have been employed during the last three hundred years, and in which mercury plays a greater or lessser part. Mr. Lee can hardly expect us to accept the two cases which he relates as a fit basis for judgment. The difficulties of employing syphilization in private practice may readily be overcome, as the experience of Christiania and other places has shown. But there is really a serious objection against many other methods of treating disease—for example, the hypodermic method of applying remedies: the doctor must see his patients often. It is certainly much more convenient, especially with hospital patients to see them once, and then send them away perhaps never to meet them again. I have seen syphilis treated in this way at the consultations in French hospitals. A patient presents himself with secondary symptoms. One rapid glance is enough. "Give him No. 14" (a preparation of the protoioduret of mercury). "Are you married?" "Yes, and my wife has the same complaint." "Give him two of No. 14." This is a very convenient manner of treating syphilis at least for the doctor, but in reality it is perhaps worse than nothing.

10. I shall finally point out a very impartial and ably written review of the progress of science with regard to the syphilitic virus. This review, "Die Lehren vom Syphilitischen Contagium, von Dr. Heinrich Auspitz Wien, 1866," contains an elaborate account of what has been done by different authors and in different countries to clear up this obscure point. The author has unconditionally adopted the views which I some years ago presented to the profession, that the soft and the indurated chancre are of common origin. Moreover, he states that the experiments on which I founded my views, especially those proving the auto-inoculability of the hard chancre, have been repeated in the service of Professor Hebra in Vienna by Dr. Pick, his assistant, and with exactly the same result as I had obtained in Christiania.

The dualistic doctrine seems after this to be doomed to the same fate as the once cherished creed, that secondary symptoms are not contagious. Like this it was started in France, and made a rapid and glorious career through Europe, but was never believed in by true observers of nature.—*London Lancet.*

Christiania, May, 1866.

